# Piracetam and Ginkgo biloba in the treatment of residual cognitive symptoms after the discontinuation of benzodiazepines in long-term users; case series and review of the literature

**DOI:** 10.1192/j.eurpsy.2023.1400

**Published:** 2023-07-19

**Authors:** T. Sugnet, S. Jonovska, V. Šendula-Jengić, P. Čargonja

**Affiliations:** 1 Department of Addictions and Psychotrauma; 2 County Hospital Insula, Rab, Croatia

## Abstract

**Introduction:**

Long-term benzodiazepine use is common although not routinely recommended. While their dependence potential is notorious, cognitive problems due to their chronic use have only recently been intensively studied. Residual cognitive symptoms may linger months after successful benzodiazepine discontinuation and significantly limit optimal functional outcome in abstinent individuals.

**Objectives:**

We present a series of cases, a 41-year-old man and a 33-year-old woman who successfully completed a ten-week benzodiazepine-dependence rehabilitation program at our hospital, including successful tapering and post-withdrawal integrative therapy. At subsequent monthly outpatient check-ups, abstinence was confirmed (including toxicology testing) and there was no residual anxiety or mood symptomatology. Still, patients complained of their suboptimal functioning due to residual cognitive problems, especially trouble concentrating and short-memory deficits, which were objectively confirmed via psychometric tests.

**Methods:**

During continuous outpatient treatment (monthly controls), patients were clinically and neurocognitively evaluated. They were reluctant to try conventional psychopharmacology agents but were open to supplement therapy. After thorough literature review, a trial of piracetam and *Ginko biloba* extract was suggested. Piracetam, a positive allosteric modulator of AMPA-receptors, has been used in the treatment of vascular neurocognitive changes in the elderly. Bilobalides from *G. biloba*, act as atypical antagonists of GABA-A receptors and were found to exert neuroprotective effects in animal models, without epileptogenic or anxiogenic risks.

**Results:**

Patients were recommended piracetam (2.4 g a day) in divided doses and a once-daily dose of *G. biloba* extract (240 mg a day), in a standardized form containing 3.2% of bilobalides. No side effects were noticed. At subsequent monthly checkups, patients reported fewer cognitive problems and better everyday functioning. Neurocognitive testing confirmed subjective findings, with positive changes in all areas, but especially so in verbal memory, which may be due to this specific pharmacological combination. At the end of the observation period of sixth months, piracetam was gradually discontinued while patients were given free choice whether to continue with G. *biloba* supplementation.

**Conclusions:**

According to our findings, piracetam and *G. biloba* extract may prove beneficial in the treatment of residual cognitive symptoms after benzodiazepine discontinuation, in long-term benzodiazepine users. Other treatments, focusing not only on modulation of glutamate and GABA, but also on other pathways should be evaluated. Further clinical studies are warranted.

**Disclosure of Interest:**

None Declared

